# Sensitive Nonenzymatic Electrochemical Glucose Detection Based on Hollow Porous NiO

**DOI:** 10.1186/s11671-017-2406-0

**Published:** 2018-01-09

**Authors:** Gege He, Liangliang Tian, Yanhua Cai, Shenping Wu, Yongyao Su, Hengqing Yan, Wanrong Pu, Jinkun Zhang, Lu Li

**Affiliations:** 1 Research Institute for New Materials Technology, Chongqing University of Arts and Sciences, Chongqing, People’s Republic of China; 2 Co-innovation Center for Micro/Nano Optoelectronic Materials and Devices, Chongqing, People’s Republic of China; 3 Faculty of Materials and Energy, Southwest University, Chongqing, People’s Republic of China

**Keywords:** NiO, Hollow porous architecture, Coordinating etching and precipitating, Electrochemical sensor, Glucose detection

## Abstract

Transition metal oxides (TMOs) have attracted extensive research attentions as promising electrocatalytic materials. Despite low cost and high stability, the electrocatalytic activity of TMOs still cannot satisfy the requirements of applications. Inspired by kinetics, the design of hollow porous structure is considered as a promising strategy to achieve superior electrocatalytic performance. In this work, cubic NiO hollow porous architecture (NiO HPA) was constructed through coordinating etching and precipitating (CEP) principle followed by post calcination. Being employed to detect glucose, NiO HPA electrode exhibits outstanding electrocatalytic activity in terms of high sensitivity (1323 μA mM^−1^ cm^−2^) and low detection limit (0.32 μM). The excellent electrocatalytic activity can be ascribed to large specific surface area (SSA), ordered diffusion channels, and accelerated electron transfer rate derived from the unique hollow porous features. The results demonstrate that the NiO HPA could have practical applications in the design of nonenzymatic glucose sensors. The construction of hollow porous architecture provides an effective nanoengineering strategy for high-performance electrocatalysts.

**Electronic supplementary material:**

The online version of this article (10.1186/s11671-017-2406-0) contains supplementary material, which is available to authorized users.

## Background

Detection of glucose with fast, accurate, and low-cost process is importance for clinical biochemistry, pharmaceutical analysis, food industry, and environmental monitoring [1–3]. Among the multitudinous techniques, electrochemical detection has been considered as one of the most convenient approach owing to its high sensitivity, low cost, and attractive lower detection limit [4–6]. However, the common glucose oxidase-based electrochemical sensors are restricted by the drawback of insufficient stability originating from the nature of enzymes [7–9]. To address these issues, earth-abundant electrocatalysts based on TMOs were recommended due to their lower cost, physicochemical stability, and redox electroactivity [10–12]. However, the overall electrocatalytic activity of conventional TMOs is still far away from the requirements of applications. It is still a challenge to rationally design high-active TMO electrocatalysts for glucose.

Generally, the process of kinetics plays a decisive role in electrocatalytic activity for established electrocatalytic materials. Inspired by the intimate connection between kinetics and microstructures, improved electrocatalytic activity can be achieved by the engineering of microstructures, including surface area, pore structure, and architecture features [13, 14]. The porous structure offers large specific surface area (SSA) and provides amounts of active sites. Furthermore, the porous structure also affords enough diffusion channels for analyte and intermediate products, which are beneficial for mass transport process [15, 16]. On the other hand, hollow structures combining functional shells and inner voids can offer larger electrolyte-electrode contact area and reduce the length for both mass and electron transport [17]. Furthermore, the available inner cavities effectively prevent electroactive nanoparticles from aggregation and accommodate the volume change and structural strain accompanied with repeated measurements [18]. In conclusion, high-active TMO electrocatalysts can be acquired through the design of hollow porous architecture.

As a typical transition metal oxide, NiO was reported as an efficient catalyst for electrooxidation of glucose due to the redox couple of Ni^3+^/Ni^2+^ in alkaline medium, implying potential applications in electrochemical glucose sensor. In this work, cubic NiO HPA was constructed through a Cu_2_O-templated coordinating etching and precipitating (CEP) method and post calcination. The hollow porous structure provides large SSA, well-defined interior voids, abundant ordered transfer channels, and high electron transfer efficiency. Being employed to detect glucose, NiO HPA electrode presents higher sensitivity and lower detection limit compared to broken NiO HPA (NiO BHPA), demonstrating advantages of the hollow porous architecture. This facile strategy to construct hollow porous architecture provides a valid method in the development of highly efficient nanomaterials for electrochemical sensors.

## Experimental

### Materials

CuCl_2_·2H_2_O, NiCl_2_·6H_2_O, Na_2_S_2_O_3_·5H_2_O, polyvinylpyrrolidone (PVP, *M*_w_ = 40,000), and NaOH were purchased from Chengdu Kelong. Glucose (Glu.), lactose (Lact.), sucrose (Sucr.), fructose (Fruc.), L-ascorbic acid (AA), uric acid (UA), and Nafion solution (5 wt% in mixture of lower aliphatic alcohols and water) were purchased from Sigma-Aldrich without further purification.

### Synthesis of Cu_2_O Template

The cubic Cu_2_O templates were synthesized according to our previous work [19]. In this typical procedure, 20 ml NaOH (2 M) was added dropwise into 200 mL CuCl_2_·2H_2_O (10 mM) under stirring at 55 °C. After 0.5 h, 4 mL AA (0.6 M) was introduced dropwise into the above solution. The suspension was further aged for 3 h and washed with water several times by centrifugation. The XRD pattern and SEM and TEM images are shown in Additional file 1: Figure S1.

### Synthesis of NiO HPA

NiO HPA was synthesized by a CEP method. First, Cu_2_O (10 mg) and NiCl_2_·6H_2_O (3 mg) were dispersed into 10 mL ethanol-water mixed solvent (volume ratio = 1:1) for 7 min by ultrasonication. Then, PVP (0.33 g) was added into the solution with vigorous agitation for 30 min. Four milliliters Na_2_S_2_O_3_ (1 M) was dropped into the system; the reaction was proceeded at room temperature for 3 h until the color of the suspension changed from red to light green. The Ni(OH)_2_ precursor was washed several times by warm ethanol-water and dried at room temperature. Finally, NiO HPA was successively obtained under an air atmosphere at 400 °C for 2 h with a slow ramp rate of 1 °C/min. NiO BHPA was obtained through strong ultrasonic treatment of NiO HPA for 2 h.

### Material Characterizations

The composition and structure of the products were characterized by X-ray diffraction (XRD, Rigaku D/Max-2400). The composition was further analyzed by the X-ray photoelectron spectroscopy (XPS, ESCALAB250Xi) with the C 1s peaks at 284.8 eV as an internal standard. The morphologies and microstructures of the products were observed using field emission scanning electron microscope (FESEM, FEI Quanta 250, Zeiss Gemini 500) and high-resolution transmission electron microscope (HRTEM, FEI F20). Brunauer-Emmett-Teller (BET, Belsort-max) was applied to analyze the specific surface area and pore structure.

### Electrochemical Measurements

All electrochemical measurements were operated in 0.1 M NaOH on μIII Autolab electrochemical workstation. A three-electrode configuration with NiO HPA (or NiO BHPA) modified glassy carbon electrode (GCE, *Ф* = 3 mm) as the working electrodes and Ag/AgCl (in saturated KCl) and platinum disk electrode (*Ф* = 2 mm) as the reference electrode and counter electrode, respectively. Typically, GCE was polished with alumina slurry (3, 0.5, and 0.05 μm). Then, the NiO HPA (10 mg) was dissolved into a mixture of 0.1 mL Nafion and 0.9 mL distilled water. Finally, 5 μL of the mixture was dropped onto the pretreated GCE (70.77 μg/cm^2^) and dried at room temperature. NiO BHPA-modified GCE was also prepared under the same condition to verify the advantages of NiO HPA. The modified electrodes were measured by cyclic voltammetry (CV), chronoamperometry (CA), and electrochemical impedance spectroscopy (EIS) to evaluate its electrocatalytic activity. EIS measurements were carried out over the frequency range between 0.01–100 kHz with a perturbation amplitude of 5 mV versus the open-circle potential.

## Results and Discussion

### Characterizations

**Fig. 1:**
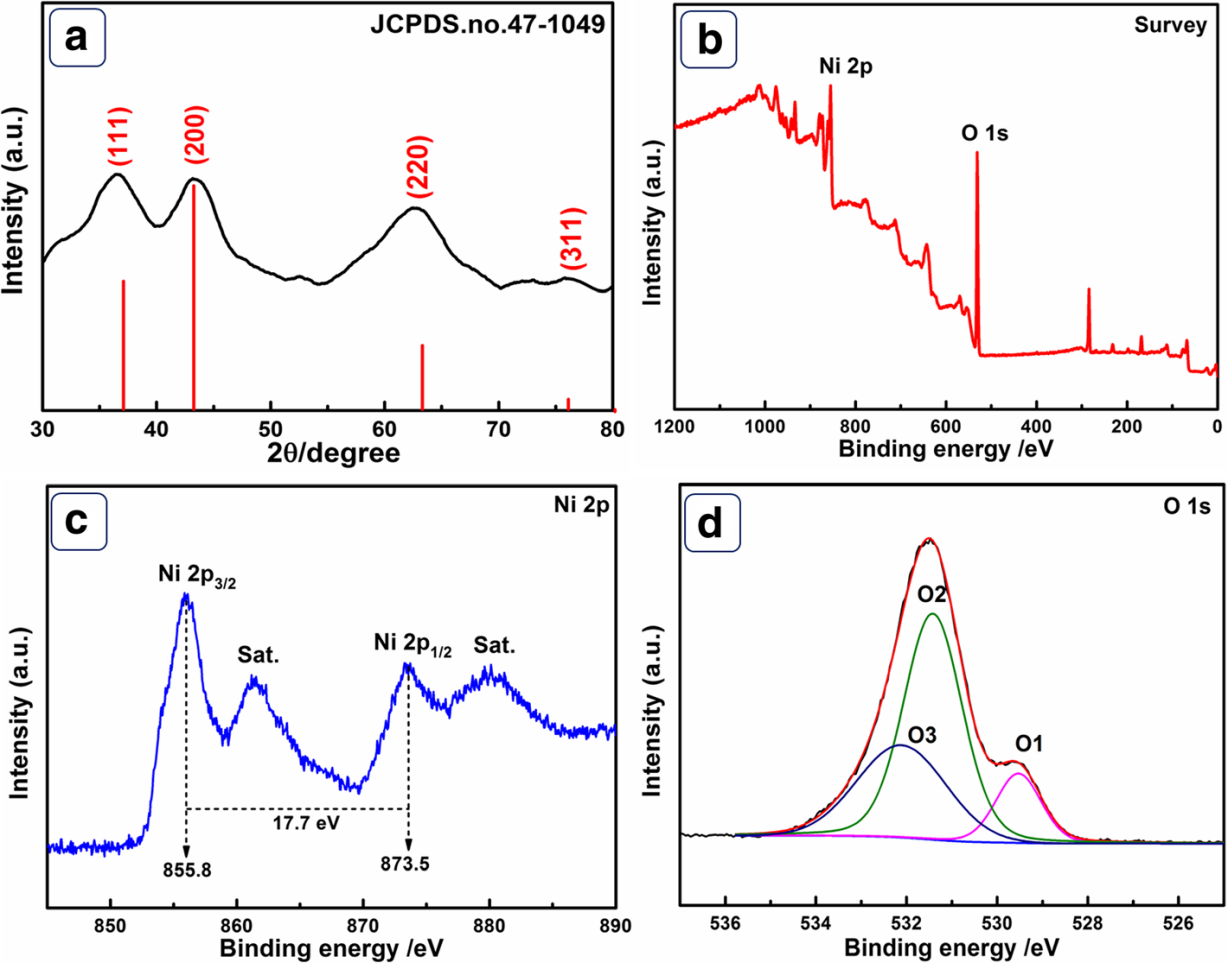
**a** XRD pattern of prepared NiO HPA. XPS spectra for the products **b** survey, **c** Ni 2p, and **d** O 1s

As shown in Fig. 1a, the diffraction peaks located at 37.21°, 43.27°, 62.87°, and 75.42° correspond to (111), (200), (220), and (311) facets of face-centered cubic NiO (JCPDS.no.47-1049) [20]. There are no other diffraction peaks, indicating the purity of the products. XPS was further employed to analyze the element composition and oxidation state of NiO HPA. The survey spectrum (Fig. 1b) demonstrates O 1s and Ni 2p peaks at 531.5 and 855.7 eV, respectively, revealing main elements of the products. In the Ni 2p spectrum (Fig. 1c, see fitting lines in Additional file 1: Table S1), two major peaks located at 855.8 eV (Ni 2p_3/2_) and 873.5 eV (Ni 2p_1/2_) with a spin-energy separation of 17.7 eV are clearly investigated, which is the feature of NiO phase [21]. The satellite peaks of Ni 2p_3/2_ and Ni 2p_1/2_ are located at around 861.5 and 880.0 eV, respectively. From Fig. 1d (see fitting lines in Additional file 1: Table S2), the fitting peak of O1 at 529.8 eV is the Ni–O bond in Ni–OH species. O2 peak at a binding energy of 831.3 eV is usually associated with chemisorbed oxygen, hydroxyls, and under-coordinated lattice oxygen. The peak of O3 at 532.7 eV is the multiplicity of physi- and chemisorbed water on/near the surface [22–24]. The analysis of XPS and XRD confirm the successful preparation of NiO.

**Fig. 2:**
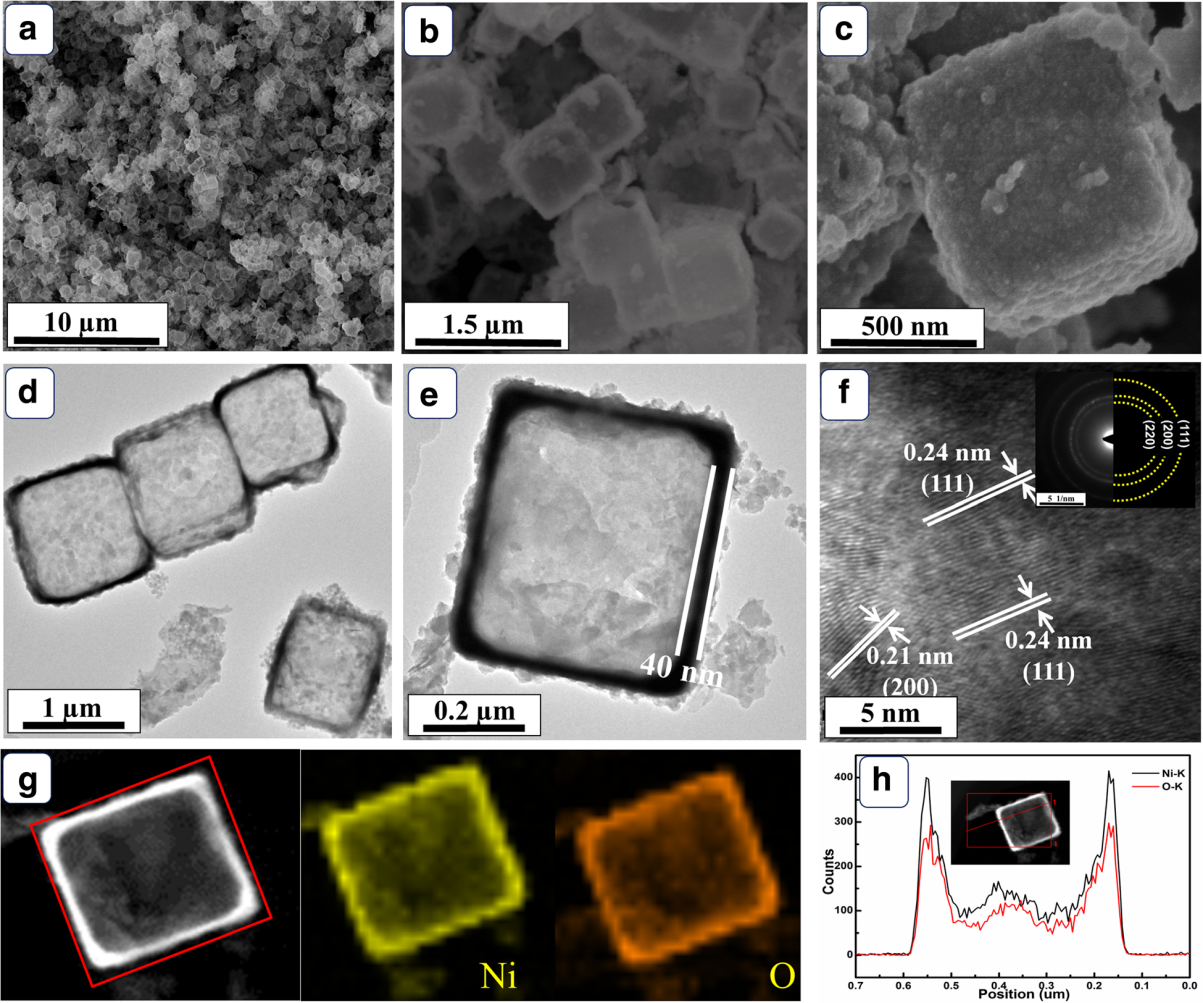
**a**–**c** SEM and **d**, **e** TEM images of NiO HPA. **f** The HRTEM image of NiO HPA. **g** The STEM and EDX mapping images of a NiO HPA cube. **h** The line-scan EDX spectra of a NiO HPA cube

The morphologies of Ni(OH)_2_ precursor (Additional file 1: Figure S2) and NiO HPA (Fig. 2) were clearly observed by SEM and TEM. The SEM images (Fig. 2a, b) of as-obtained NiO present uniform cubic feature with an edge length about 600 nm. From Fig. 2c, it is clearly observed that the rough shell of NiO HPA consists of amounts of interconnected fine particles. As shown in Fig. 2d, the border of NiO products is black and the interior is translucent. Combing with the SEM observations in Fig. 2a–c, the cubic hollow characteristics of the NiO products can be confirmed. As displayed in Fig. 2e, the shell thickness of the cube is about 40 nm, which is thinner than that of Ni(OH)_2_ precursor (about 60 nm). The shrink of shell thickness is attributed to the loss of H_2_O in the precursor after heat treatment. In Fig. 2f, the spacing for marked adjacent lattice fringes are about 0.21 and 0.24 nm, respectively, corresponding to (200) and (111) facets of NiO. The selected area electron diffraction (SAED) rings can be indexed to (111), (200), and (220) facets of NiO inside and out, which agrees well with the XRD results [25]. In addition, the elemental mapping images in Fig. 2g exhibit surface rich distribution of Ni and O. As shown in Fig. 2h, the line-scan EDX profile demonstrates the uniform near-surface distribution of O and Ni, reconfirming the hollow architecture. NiO HPA would provide enough active sites and abundant diffusion channels, which favor the mass transfer process for electrolyte and glucose. Furthermore, the thin shell of NiO HPA apparently shortens the transfer distance of electrons and accelerates the transfer rate, endowing NiO HPA with promising electrocatalytic activity.

**Fig. 3:**
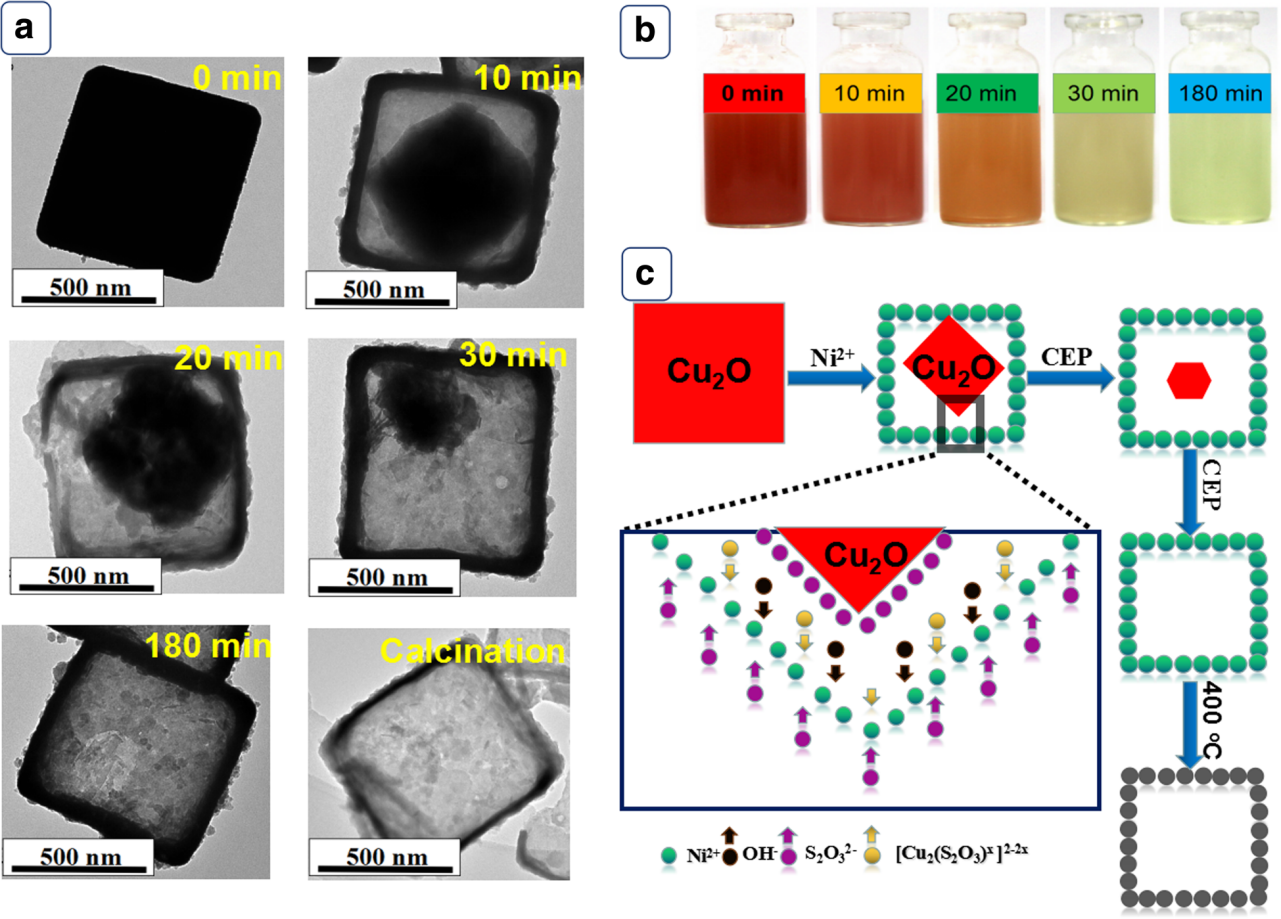
**a** TEM images of the products monitored at different reaction times. **b** Optical photographs of the suspension at different reaction time after addition of etchant. **c** Schematic illustration of the proposed growth mechanism of NiO HPA

In order to understand the relevant formation mechanism, the precipitate prepared at 0, 10, 20, 30, and 180 min were collected and observed by TEM. As shown in Fig. 3a, the solid cubic Cu_2_O crystal has an edge length about 600 nm. With the introduction of S_2_O_3_^2−^, the coordinating etching of Cu_2_O preferentially occurs at the corner due to higher diffusion intensity [26]. As the reaction proceed, the interior Cu_2_O templates significantly shrink to octahedron-like structure until completely removed. As observed in Fig. 3b, the color of the reaction system gradually becomes shallow and the light green precipitates generate at the same time. Combined with TEM results, the overall CEP route and formation mechanism were illustrated in Fig. 3c. The CEP mechanism can be described as follows: (i) Cu^+^ prefers to form soluble [Cu_2_(S_2_O_3_^2−^)_*x*_]^2 − 2*x*^ complex through the combination with S_2_O_3_^2−^ (reaction (1)) and simultaneously OH^−^ is released; (ii) The partly hydrolyzation of S_2_O_3_^2−^ promotes the supply of OH^−^ (reaction (2)). (iii) Reactions (1) and (2) synchronously drive reaction (3) from left to right, facilitating the formation of Ni(OH)_2_ shell [27]. Regarding kinetics factors, the etching rate of Cu_2_O depends on the diffusion of S_2_O_3_^2−^ from exterior into internal space and the growth rate of Ni(OH)_2_ shell is correlated to the transport of OH^−^ from interior to exterior [28]. Synchronously controlling of etching rate towards Cu_2_O and precipitating rate of Ni(OH)_2_ shell leads to the formation of well-defined hollow Ni(OH)_2_ precursor. NiO HPA is finally obtained through the post calcination of Ni(OH)_2_ precursor.

1 $$ {\mathrm{Cu}}_2\mathrm{O}+x{\mathrm{S}}_2{\mathrm{O}}_3^{2-}+{\mathrm{H}}_2\mathrm{O}\to {\left[{\mathrm{Cu}}_2{\left({\mathrm{S}}_2{\mathrm{O}}_3\right)}_x\right]}^{2-2x}+2{\mathrm{O}\mathrm{H}}^{-} $$

2 $$ {\mathrm{S}}_2{\mathrm{O}}_3^{2-}+{\mathrm{H}}_2\mathrm{O}\rightleftharpoons {\mathrm{H}\mathrm{S}}_2{\mathrm{O}}_3^{2-}+{\mathrm{O}\mathrm{H}}^{-} $$

3 $$ {\mathrm{Ni}}^{+}+2{\mathrm{OH}}^{-}\to \mathrm{Ni}{\left(\mathrm{OH}\right)}_2 $$

**Fig. 4:**
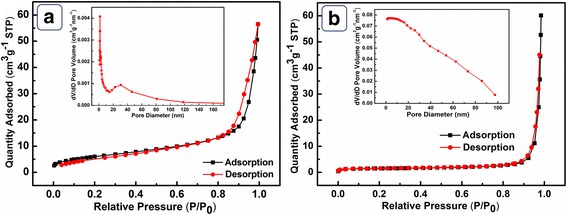
N_2_ adsorption-desorption isotherms of **a** NiO HPA and **b** NiO BHPA. Inset of **a** and **b** are the corresponding pore size distribution, respectively

The surface area and porosity of NiO HPA and NiO BHPA (Additional file 1: Figure S3) were also characterized by BET method. NiO HPA possesses SSA of 27.08 m^2^/g and a pore volume of 0.087 cm^3^/g (Fig. 4a), which is much larger than the reported NiO materials [29]. Regarding the pore size distribution, NiO HPA mainly presents a concentrated distribution at around 7 nm, which is related to the ordered channels between NiO nanoparticles. The large SSA and ordered channels can effectively improve the absorption of analyte and mass transport process, leading to enhanced electrocatalytic activity. The SSA and pore volume of the broken sample are 5.24 m^2^/g and 0.078 cm^3^/g (Fig. 4b), respectively, which is much smaller than those of NiO HPA. This can be attributed to the collapse of original hollow structure after ultrasonic treatment. Notably, no concentrated pore distribution is observed for NiO BHPA (inset of Fig. 4b), indicating complete destruction of ordered diffusion channels. The decrease of SSA and destruction of ordered diffusion channels are adverse for kinetics, which may result in poor electrocatalytic activity. Accordingly, NiO HPA possesses beneficial microstructures for electrocatalysis compared to the broken samples.

### Electrochemical Performance

**Fig. 5:**
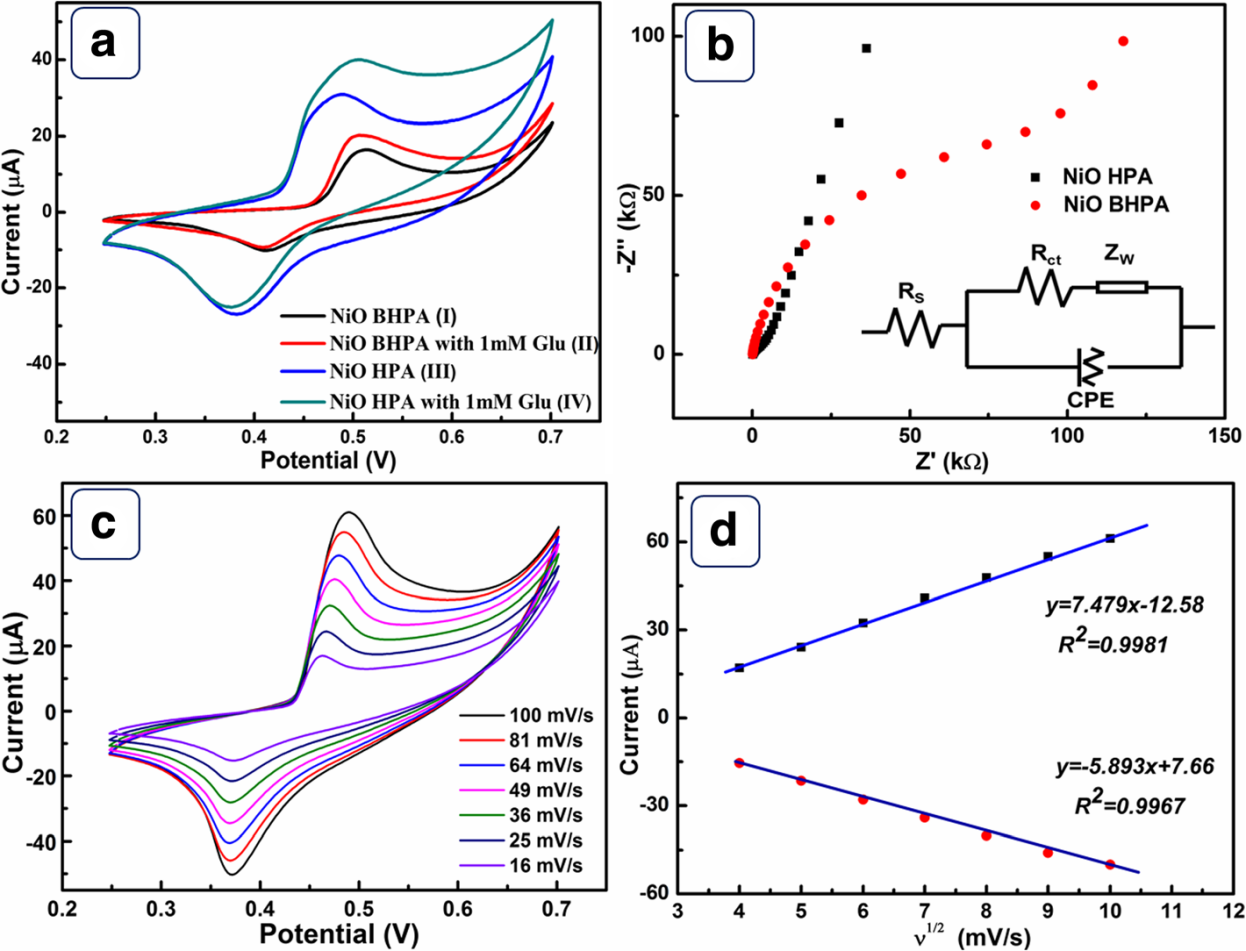
**a** CVs of NiO BHPA (I, II) and NiO HPA (III, IV) electrode with (II, IV) and without (I, III) the presence of 1 mM glucose in 0.1 M NaOH at scan rate 50 mV/s. **b** Nyquist diagrams EIS and equivalent circuit of NiO HPA and NiO BHPA in 0.1 M NaOH solution. **c** CVs of NiO HPA electrode at various scan rates in 0.1 M NaOH with 1 mM glucose and **d** the relationship between peak current and square root of scan rates

Figure 5a shows the CVs of NiO HPA and NiO BHPA electrodes with and without 1 mM glucose. A pair of well-defined peaks located at 0.48 and 0.38 V are clearly investigated in curve III, which are related to Ni^2+^/Ni^3+^ redox couple. The redox peak current of curve III is obviously higher than that of curve I. This is related to the collapse of hollow architecture and the decrease of SSA. Upon the addition of glucose, current responses are clearly observed on both electrodes (curve II and IV). NiO HPA electrode exhibits higher current response than that of NiO BHPA electrode. In addition, the onset potential for electrooxidation of glucose on NiO HPA electrode (0.43 V) is lower than that of NiO BHPA electrode (0.46 V), revealing higher electrocatalytic activity. The high electrocatalytic activity is attributed to large amounts of active sites, ordered pore structure, and high electron transfer rate provided by the hollow porous structure. The electrooxidation of glucose on NiO HPA electrode is driven by Ni^2+^/Ni^3+^ redox couple in alkaline medium according to the following reactions [30, 31]:

4 $$ \mathrm{NiO}\to {\mathrm{Ni}}^{2+}+{\mathrm{O}}^{2-} $$

5 $$ {\mathrm{Ni}}^{2+}+{\mathrm{OH}}^{-}\to {\mathrm{Ni}}^{3+}+{e}^{-} $$

6 $$ {\mathrm{Ni}}^{3+}+\mathrm{glucose}\to {\mathrm{Ni}}^{2+}+\mathrm{gloconic}\  \mathrm{acid} $$

As shown above, OH^−^ plays an important role in the electrocatalytic reaction. Obviously, alkaline medium accelerates the redox of Ni^2+^/Ni^3+^ compared to neutral medium (Additional file 1: Figure S4), leading to higher electrocatalytic activity.

Nyquist plots of NiO HPA and NiO BHPA electrodes were displayed in Fig. 5b. Each plot is characterized by a semicircle in the high-frequency region and a straight line in the low-frequency region. Generally, the intercept on the real axis represents the solution resistance (*R*_s_), which is composed of intrinsic resistance, ionic resistance, and contact resistance. The semicircle diameter related to electron transfer resistance is represented by *R*_ct_. As shown in Additional file 1: Table S3, NiO HPA electrode exhibits smaller *R*_s_ and *R*_ct_ than NiO BHPA. The facts can be attributed to the beneficial electron transfer kinetics derived from the hollow feature. The slope of the impedance plot in the low frequency range corresponds to the Warburg impedance (*Z*_w_), which represents the diffusive resistance [32]. It is clear that NiO HPA favors the diffusion kinetics; however, the NiO BHPA hinders the diffusion of electrolyte. This can be ascribed to the destruction of the ordered diffusion channels after ultrasonic. On the basis of above EIS discussions, NiO HPA electrode is more beneficial for both electron and mass transfer kinetics compared to the broken sample, implying the advantages of NiO HPA as an electrocatalyst for glucose.

The kinetics of NiO HPA electrode was determined from the CVs with different scan rates in 1 mM glucose solution (Fig. 5c). As depicted in Fig. 5d, the anodic and cathodic peak currents are proportional to the square root of scan rates, demonstrating a typical diffusion-controlled dynamic process. Furthermore, no significant positive/negative shift is observed for anodic/cathodic peak, implying unimpeded diffusion kinetics originated from the hollow porous structure.

### The Selectivity, Reproducibility, and Stability of NiO HPA Electrode

**Fig. 6:**
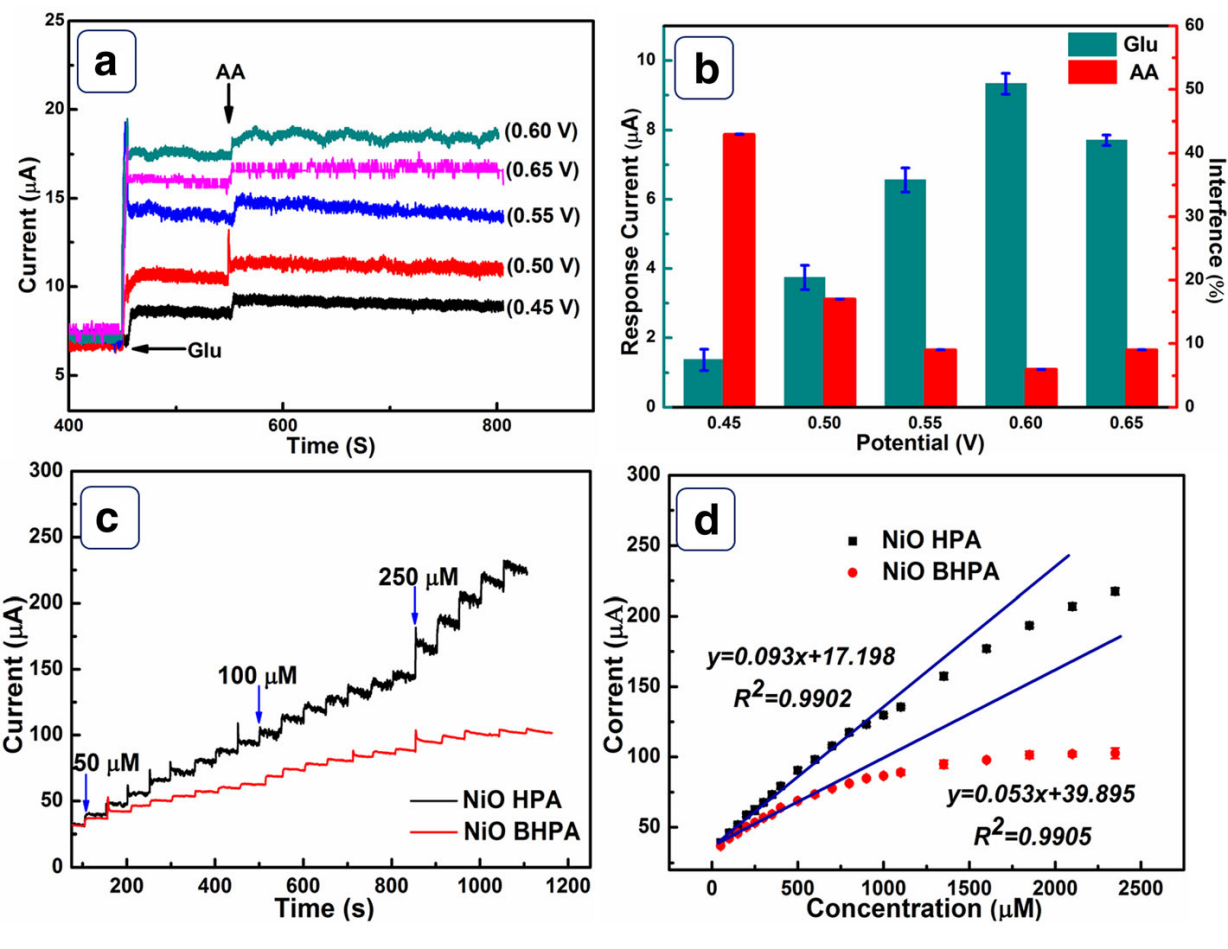
**a** Amperometric response of NiO HPA electrode at different potentials with the addition of 0.1 mM glucose and 0.01 mM AA. **b** The response current of glucose and AA at different potentials. **c** CA of NiO HPA and NiO BHPA electrode at 0.6 V with the successive addition of glucose. **d** The relationship between response current and the concentration of glucose

To obtain optimized working potential, current response of glucose and interference of AA were taken into consideration under different potentials and the data were displayed in Fig. 6a. From the statistical data in Fig. 6b, 0.6 V was selected by the fact that NiO HPA electrode exhibits maximum current response to glucose and minimum interference to AA at 0.6 V. Figure 6c displays the typical amperometric responses of NiO HPA and NiO BHPA towards different concentration of glucose at 0.6 V. Notable current responses are clearly observed for the two electrodes, and the current responses increase with the glucose concentration increasing. Figure 6d presents the relationship between response currents and glucose concentration for NiO HPA and NiO BHPA electrodes. NiO HPA electrode presents a linear range from 0.32 to 1100 μM with a sensitivity of 1323 μA mM^−1^ cm^−2^, which is higher than that of NiO BHPA electrode (753 μA mM^−1^ cm^−2^). Moreover, the limit of detection (LOD) of NiO HPA electrode (0.32 μM) is much lower than that of NiO BHPA (14.2 μM). To manifest the advantages of NiO HPA, the performance of NiO HPA electrode was compared with other reported NiO-based glucose detection electrodes in Table 1. It is found that NiO HPA electrode presents satisfying electrocatalytic activity towards glucose in terms of high sensitivity and low LOD, indicating potential applications in electrochemical glucose sensors. This is essentially attributed to the abundant active sites, faster mass transport kinetics, and accelerated electron transfer kinetics derived from the highly porous hollow architecture.

**Table 1:** Comparison of researched electrode with reported nonenzymatic glucose sensors based on NiO

Electrode	Sensitivity (μA mM^−1^ cm^−2^)	Linear range (mM)	LOD (μM)	Reference
NiO HPA/GCE	1323	0.0025–1.10	0.32	This work
NiO/GCE	67.34	0.076–3.0	25.35	[38]
Pt/NiO/ERGO^a^/GCE	668.2	0.002-5.66	0.2	[39]
Hedgehog-like NiO	1052.8	0.1–50 (μM)	1.2	[40]
Pt–NiO nanofiber/GCE	180.8	Up to 3.67	0.313	[41]
Ag/NiO nanofibers	19.3	Up to 0.59	1.37	[42]
NiO–Ag nanofiber/GCE	170	Up to 2.63	0.72	[43]
NiO hollow nanospheres	343	1500–7000	47	[44]
NiO–CdO nanofiber/GCE	212.71	Up to 6.37	0.35	[45]
Cu/NiO nanocomposites	171.8	0.5–5	0.5	[46]

^a^Electrochemically reduced graphene oxide

**Fig. 7:**
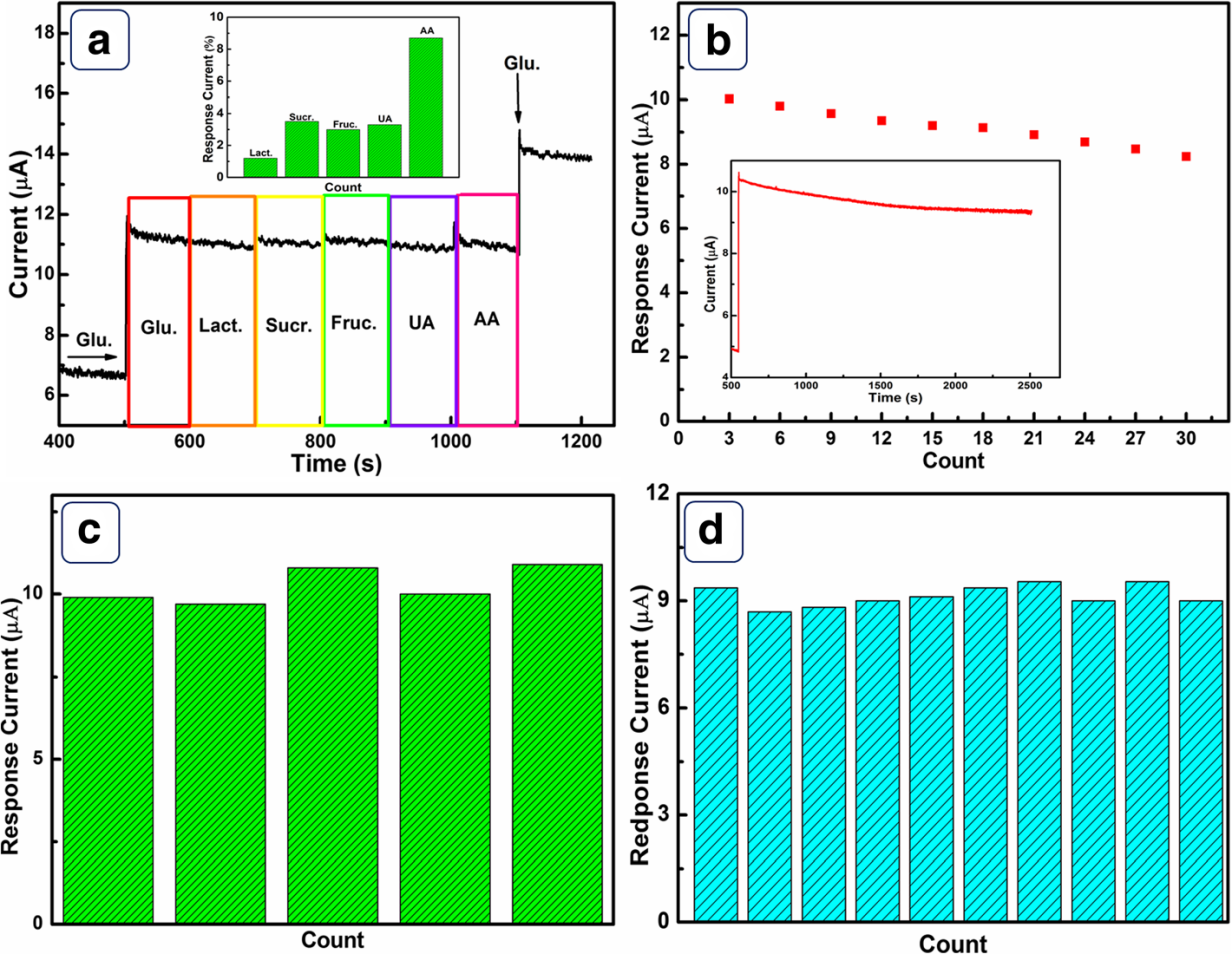
**a** The current response of NiO HPA electrode to sequential addition of 50 μM glucose and 5 μM interfering species at an applied potential of 0.6 V. Inset is the statistical data of the interference current. **b** Long-term stability of NiO HPA electrode for 0.1 mM glucose. Inset is the stability of NiO HPA electrode with the running time. **c** Current responses of five NiO HPA electrodes towards 0.1 mM glucose. **d** Ten measurements of a NiO HPA electrode towards 0.1 mM glucose

Selectivity is an important indicator to assess the performance of glucose sensors. Some easily oxidized compounds, such as Lact., Sucr., Fruc., UA, and AA normally co-existed with glucose in human blood. Notably, the physiological level of these interfering species is more or less one tenth of the glucose concentration [33]. Thus, the selectivity of NiO HPA electrode was evaluated by introducing 0.01 mM above interfering species during amperometric measurement towards 0.1 mM glucose. As shown in Fig. 7a, no severe interference is observed for Lact., Sucr., Fruc., and UA. The major interfering species AA only exhibit 8.7% interference current towards glucose. Furthermore, the second addition of 50 μM glucose still retains about (89 ± 0.2)% of its original response, indicating excellent anti-interference performance. The outstanding selectivity could be attributed to the electrostatic repelling effect between NiO HPA electrode and interfering species. NiO HPA electrode would be negatively charged in 0.1 M NaOH because the pH of electrolyte is above the isoelectric point of NiO [34]. In addition, the major interfering species (AA) is easy to lose protons in alkaline solution and possess a negatively charged shell [35]. The electrostatic repulsion between the shell of interferent and NiO HPA electrode leads to improved selectivity. The stability of NiO HPA electrode was estimated by measuring its current responses towards 0.1 mM glucose over 30 days. In Fig. 7b, the current response still retains 83.13% of its initial response after 30 days, revealing excellent long-term stability of NiO HPA electrode at room temperature. The current response of NiO HPA electrode towards 0.1 mM glucose is stable over an operation time of 2000 s with a loss of 9.82% of its original response. The five independently prepared NiO HPA electrodes exhibit an acceptable RSD of 3.12% for current responses towards 0.1 mM glucose at 0.6 V. Moreover, current responses for a same NiO HPA electrode towards 0.1 mM glucose were measured for ten times and the current responses display a RSD of 2.36%, demonstrating remarkable reproducibility. The NiO HPA electrode expresses high sensitivity, excellent stability, and remarkable reproducibility, making it attractive for practical applications.

### Detection of Glucose in Human Serum

NiO HPA electrode was further applied to detect glucose level in human blood, and the results were compared with a medical equipment (Table 2). The serums samples were provided by a local hospital and diluted with alkaline electrolytes before measurements [36, 37]. The response current measured at 0.6 V was recorded to calculate corresponding glucose concentration according to working equation. NiO HPA electrode shows a RSD of 2.85% towards detection of glucose. In addition, NiO HPA electrode presents accredited recovery between 92 and 102%, demonstrating excellent practicability in the determination of glucose in human serum.

**Table 2:** Detection of glucose in human serum

Sample	Measured by medical equipment (mM)	Measured by NiO (mM)	RSD (%)	Added (mM)	After adding (mM)	Recovery (%)
1	3.6	3.5	2.85	5.0	8.4	98
2	5.1	5.2	2.93	5.0	9.8	92
3	7.6	7.5	3.84	5.0	12.6	102

All the concentration tests and RSD calculations are of five independent measurements

*Recovery* = (after adding − before adding)/added × 100%

## Conclusions

In summary, we have successfully fabricated a NiO HPA electrocatalyst for glucose through a CEP method. The NiO HPA offers large SSA, ordered pore structure, and short electronic transfer route, which are beneficial for electrocatalytic kinetics. As a nonenzymatic glucose detection electrode, NiO HPA exhibits higher sensitivity of 1323 μA mM^−1^ cm^−2^ and lower LOD of 0.32 μM compared to NiO BHPA. In the term of selectivity, less than 8.7% interference is investigated for the common interfering species. Simultaneously, NiO HPA electrode retains 89.02% of its original response after 30 days. In addition, the designed NiO HPA was successfully applied to detect glucose in human serum. NiO HPA presents accredited stability and practicability compared to medical equipment. The design of hollow porous architecture paves a high efficient way to obtain low cost and high-performance electrocatalysts for glucose.

## Additional file

Additional file 1: Supplementary figures and tables. (DOC 6924 kb)
