# Supplementary material for: Sensitive Nonenzymatic Electrochemical Glucose Detection Based on Hollow Porous NiO
Source: Nanoscale Res Lett. 2018 Jan 9;13:3. doi: 10.1186/s11671-017-2406-0 (PMC5760490; doi:10.1186/s11671-017-2406-0)
Supplement: Additional file 1: — Supplementary figures and tables. (DOC 6924 kb) [file 11671_2017_2406_MOESM1_ESM.doc]

**Sensitive** **non-enzymatic** **electrochemical glucose detection based on hollow porous NiO**

**Supporting Information**

Gege He1,2,3, Liangliang Tian1,2,*, Yanhua Cai1,2, Shenping Wu1, Yongyao Su1,2, Hengqing Yan1,2, Wanrong Pu1,2, Jinkun Zhang1,2 and Lu Li1,2,*

1Research Institute for New Materials Technology, Chongqing University of Arts and Sciences, Chongqing, PR China

2 Co-innovation Center for Micro/Nano Optoelectronic Materials and Devices

3 Faculty of Materials and Energy, Southwest University, Chongqing, PR China

E-mail addresses: tianll07@163.com (LL Tian), lli@cqwu.edu.cn (L Li)

**This document file includes:**

Supplementary Figure S1 to S4

Supplementary text

Supplementary Table S1 to S3


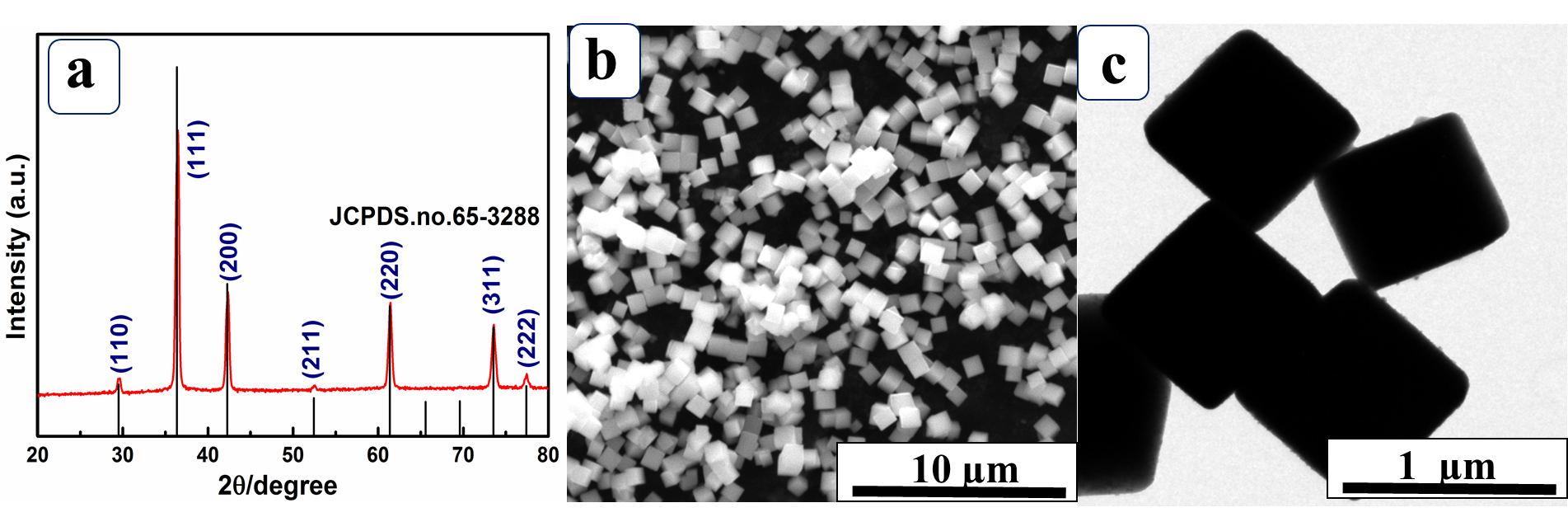


Figure S1. (a) XRD pattern of the prepared Cu­2O templates; (b) SEM and (c) TEM images of Cu2O templates.

The phase and structure of prepared Cu2O were determined by XRD. The diffraction peaks of Cu2O match well with JCPDS no.65-3288, confirming the successful preparation of Cu2O (Fig. S1a). As shown in Fig. S1b and c, Cu2O templates present well defined solid cubic features with an edge length about 600 nm.


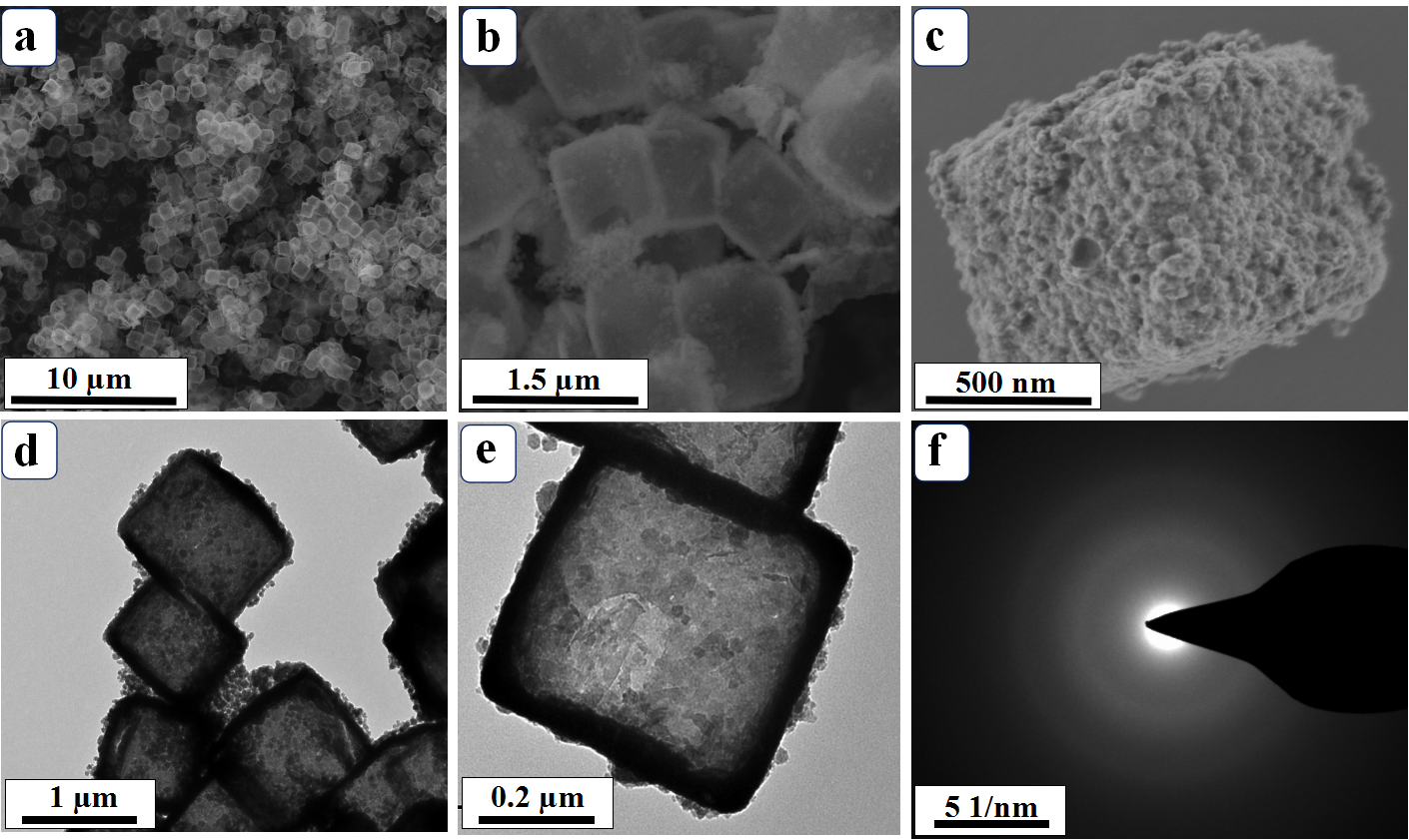


Figure S2. (a, b, c) SEM and (d, e) TEM images of the Ni(OH)2 precursor; (f) SAED of the Ni(OH)2 precursor.

The surface morphology of Ni(OH)2 precursor were investigated by SEM and TEM. Fig. S2a-b show uniform cubic features of the products with an edge length about 600 nm. From Fig. S2c. the rough surface of Ni(OH)2 is composed of a large number of interconnected nanoparticle­­s. Further insight into the structure, the TEM images of Ni(OH)2 precursor confirm the hollow architecture with a shell thickness of about 60 nm (Fig. S2d-e). The SAED pattern in Fig. S2f demonstrates the amorphous essence of Ni(OH)2 precursor.


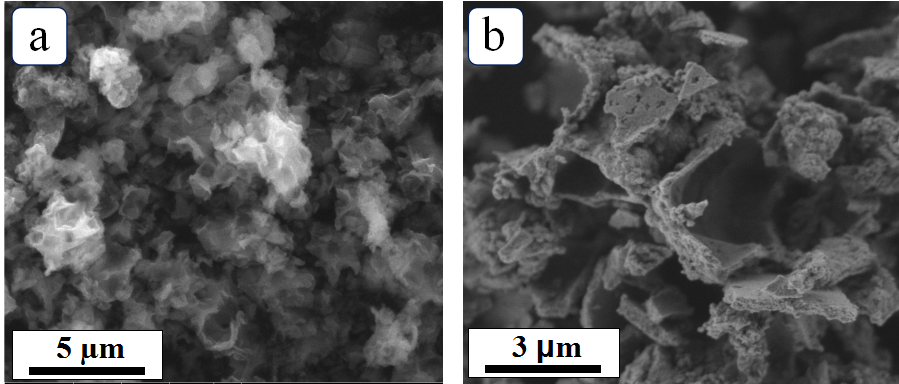


Figure S3. (a, b) SEM images of the NiO BHPA


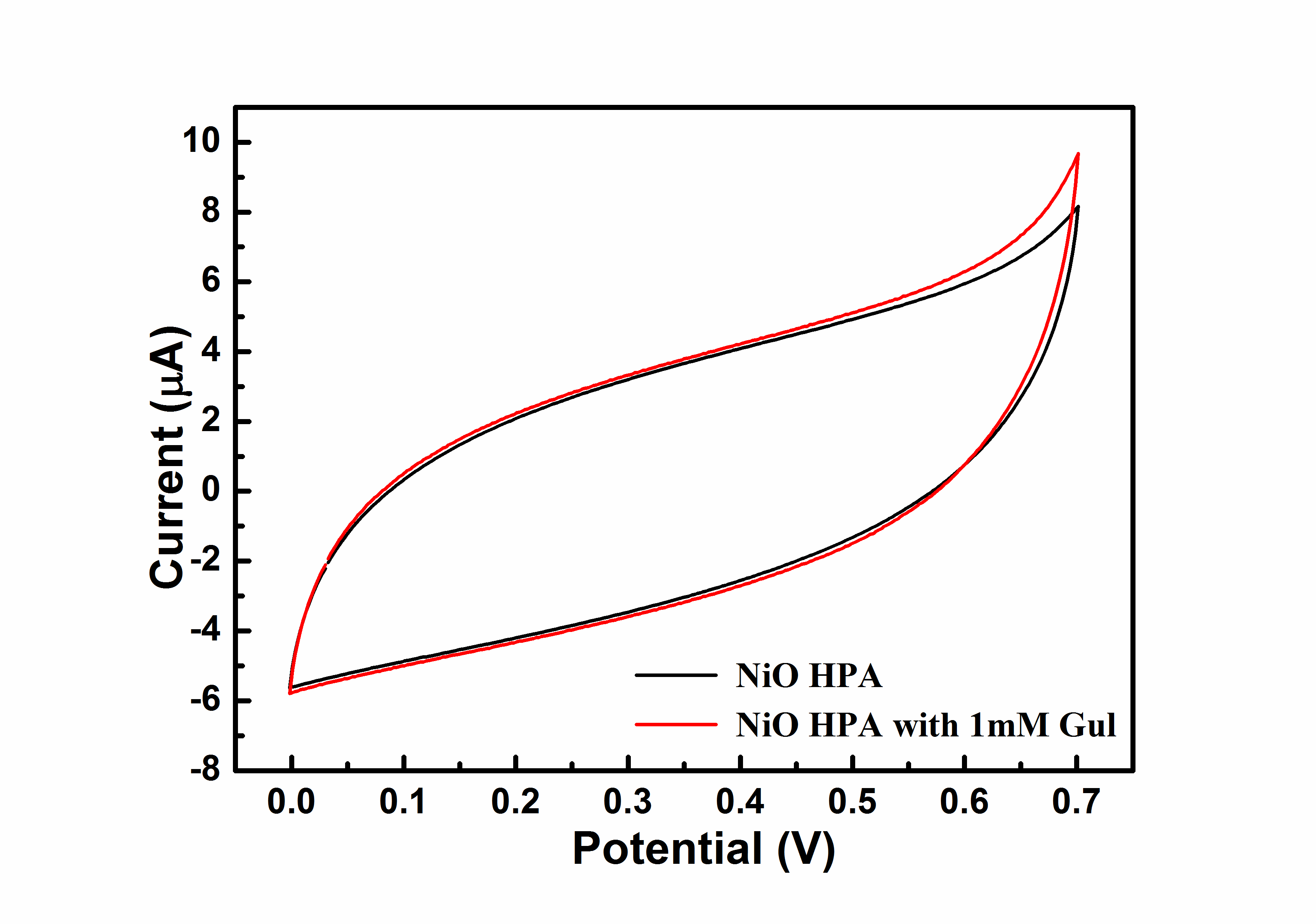


Figure S4. CVs of NiO HPA electrode with and without the presence of 1 mM glucose in 0.1 M PBS at scan rate 50 mV/s

**Table S1. The XPS fitting lines for Ni 2p.**

| Peak | Position (eV) | FWHMa (eV) | Area |
| --- | --- | --- | --- |
| Ni 2p3/2 | 855.8 | 3.4 | 64008.2 |
| Ni 2p1/2 | 873.5 | 3.2 | 25087.4 |
| Sat. (Ni 2p3/2) | 861.8 | 4.6 | 40228.2 |
| Sat. (Ni 2p1/2) | 880.0 | 6.4 | 37749.1 |

a Full width half maximum

**Table S2. The XPS fitting lines for O 1s.**

| Peak | Position (eV) | FWHMa (eV) | Area |
| --- | --- | --- | --- |
| O1 | 529.8 | 1.1 | 12146.5 |
| O2 | 531.3 | 1.5 | 51929.3 |
| O3 | 532.7 | 2.3 | 29636.9 |

a Full width half maximum

Table S3. Comparison of researched NiO HPA electrode with broken NiO HPA about EIS.

| Samples | NiO BHPA | NiO HPA |
| --- | --- | --- |
| *Rs* | 141 Ω | 77.7 Ω |
| *Rct* | 126 KΩ | 7.73 KΩ |
| *Zw* | 19.0 µMho (N=0.776) | 58.9 µMho (N=0.799) |

*The sample of EIS data estimated error less than 5%.
